# The effect of systematic pediatric care on neonatal mortality and hospitalizations of infants born with oral clefts

**DOI:** 10.1186/1471-2431-11-121

**Published:** 2011-12-28

**Authors:** George L Wehby, Eduardo E Castilla, Norman Goco, Monica Rittler, Viviana Cosentino, Lorette Javois, Mark Kindem, Hrishikesh Chakraborty, Graca Dutra, Jorge S López-Camelo, Iêda M Orioli, Jeffrey C Murray

**Affiliations:** 1 College of Public Health, Department of Health Management and Policy, University of Iowa, Iowa City, IA, USA; 2 ECLAMC (Latin American Collaborative Study of Congenital Malformations) at Cemic, Buenos Aires, Argentina; ECLAMC at Instituto Oswaldo Cruz, Rio de Janeiro, Brazil; ECLAMC at INAGEMP: National Institute of Population Medical Genetics; 3 Statistic Research Division, RTI International, Research Triangle Park, NC, USA; 4 ECLAMC at Maternidad Ramón Sardá, Buenos Aires, Argentina; 5 ECLAMC at Cemic, Buenos Aires, Argentina; 6 Developmental Biology, Genetics and Teratology Branch, Center for Developmental Biology and Perinatal Medicine, Eunice Kennedy Shriver National Institute of Child Health and Human Development, National Institutes of Health, Bethesda, MD, USA; 7 Statistic Research Division, RTI International, Research Triangle Park, NC, USA; 8 Department of Epidemiology and Biostatistics, Arnold School of Public Health The University of South Carolina, Columbia, SC, USA; 9 ECLAMC at Instituto Oswaldo Cruz, Rio de Janeiro, Brazil; ECLAMC at INAGEMP: National Institute of Population Medical Genetics; 10 ECLAMC at Imbice, La Plata, Argentina; ECLAMC (Latin American Collaborative Study of Congenital Malformations) at Cemic, Buenos Aires, Argentina; 11 ECLAMC at Departamento de Genética, Universidade Federal do Rio de Janeiro, Brazil; ECLAMC at INAGEMP: National Institute of Population Medical Genetics; 12 College of Medicine, Department of Pediatrics, University of Iowa, Iowa City, IA, USA

## Abstract

**Background:**

Cleft lip and/or palate (CL/P) increase mortality and morbidity risks for affected infants especially in less developed countries. This study aimed at assessing the effects of systematic pediatric care on neonatal mortality and hospitalizations of infants with cleft lip and/or palate (CL/P) in South America.

**Methods:**

The intervention group included live-born infants with isolated or associated CL/P in 47 hospitals between 2003 and 2005. The control group included live-born infants with CL/P between 2001 and 2002 in the same hospitals. The intervention group received systematic pediatric care between the 7^th ^and 28^th ^day of life. The primary outcomes were mortality between the 7^th ^and 28^th ^day of life and hospitalization days in this period among survivors adjusted for relevant baseline covariates.

**Results:**

There were no significant mortality differences between the intervention and control groups. However, surviving infants with associated CL/P in the intervention group had fewer hospitalization days by about six days compared to the associated control group.

**Conclusions:**

Early systematic pediatric care may significantly reduce neonatal hospitalizations of infants with CL/P and additional birth defects in South America. Given the large healthcare and financial burden of CL/P on affected families and the relatively low cost of systematic pediatric care, improving access to such care may be a cost-effective public policy intervention.

**Trial Registration:**

ClinicalTrials.gov: NCT00097149

## Background

Cleft lip and/or palate (CL/P) are common and burdensome birth defects, occurring in about 1 in 700 births, with variation by ethnicity and socioeconomic status [1]. CL/P occur in isolation or in association with other birth defects. The isolated forms are a result of a complex etiology of genetic and environmental factors [2-8] The associated forms occur due to various etiologies involving more than 500 Mendelian disorders, chromosome anomalies, teratogens and uncategorized syndromes [9]. CL/P may result in significant early life problems including feeding problems, surgery-related events, airway management and infections [10]. These complications increase early life mortality risk, particularly in associated forms [10-14]. CL/P are among the most common birth defects, and neonatal mortality from birth defects is a significant impediment to reducing under-five mortality as part of the Millennium Development Goals effort [15]. Further, CL/P significantly increase hospitalization use and costs during early life and childhood [16,17]. In the United States (US), first-year inpatient healthcare expenditures for infants with CL/P are about five times higher than unaffected children, with higher expenditures by more than eight times among associated compared to isolated cases [17]. CL/P also increase total healthcare expenditures during the first ten years of life by about eight times [16]. In addition to these early-life impacts, CL/P increase the risks of mortality [18], mental health problems [19] and certain cancers [20] later in life. CL/P may reduce the quality of life of affected individuals and families [21] in both isolated and associated cases and result in psychological and social adjustment problems [22-25].

Systematic pediatric care plays a key role in addressing the early life complications of CL/P. Access to such care during the neonatal period is critical for identifying and treating complications and educating parents in feeding and other care practices. There have been no large-scale studies of CL/P impacts on mortality and hospitalizations in less developed countries and of the effects of systematic pediatric care on these risks. This study assesses the effects of a systematic pediatric care program on neonatal mortality and hospitalizations of infants with CL/P in South America. The study background and methods have been described in greater detail elsewhere [26]. This paper reports the intervention effects on mortality and hospitalization use.

## Methods

### Setting and Participants

The study intervention population included a consecutive series of in-hospital live-born infants with any form of isolated or associated typical CL/P, between January 1, 2003 and December 31, 2005 in 47 hospitals in Argentina, Brazil, Bolivia, Chile, Colombia, Ecuador and Venezuela [26]. The isolated group had no other major birth defects besides CL/P and no syndromes including those with or without chromosomal abnormalities. Pierre-Robin was considered as isolated cleft palate. Associated cases were identified using physical exams and medical expert review [27]. The control population included all infants with typical CL/P born between January 1, 2001 and December 31, 2002 in the same study hospitals as the intervention group. The same definitions for isolated and associated status were used for the treatment and control groups. Table 1 reports the distribution of the other birth defects in the associated treatment and control groups. The investigators considered randomizing the prospectively enrolled group into control and intervention groups to be unethical given that the study's intervention represents the standard of care in developed countries such as the United States, where early and regular evaluation of infants with oral clefts within the neonatal period is emphasized [28].

**Table 1:** Distribution of Birth Defects in the Treatment and Control Associated Groups

Birth Defect Type	Control group		Treatment group		Total study sample	
	
	Frequency	Percent	Frequency	Percent	Frequency	Percent
Abdominal wall defect	7	1.88	5	1.04	12	1.40
Anencephaly	9	2.41	5	1.04	14	1.64
Spina bifida	4	1.07	2	0.41	6	0.70
Brain defect	28	7.51	41	8.49	69	8.06
Cephalocele	3	0.80	8	1.66	11	1.29
Eye anomaly	14	3.75	22	4.55	36	4.21
Microtia	10	2.68	23	4.76	33	3.86
Congenital heart defect	32	8.58	48	9.94	80	9.35
Lung hypoplasia	1	0.27	2	0.41	3	0.35
Esophageal atresia	3	0.80	4	0.83	7	0.82
Gut anomaly	2	0.54	3	0.62	5	0.58
Genitalia defect	13	3.49	15	3.11	28	3.27
Kidney defect	7	1.88	23	4.76	30	3.50
Talipes	27	7.24	31	6.42	58	6.78
Polydactyly	26	6.97	17	3.52	43	5.02
Syndactyly	10	2.68	8	1.66	18	2.10
Limb reduction defect	7	1.88	17	3.52	24	2.80
Hip dislocation	1	0.27	2	0.41	3	0.35
Arthrogryposis	7	1.88	4	0.83	11	1.29
Axial defect	10	2.68	3	0.62	13	1.52
Hydrops	2	0.54	0	0.00	2	0.23
Situs inversus	1	0.27	0	0.00	1	0.12
Syndromes	24	6.43	17	3.52	41	4.79
Minor anomalies	90	24.13	143	29.61	233	27.22
Other anomalies	35	9.38	40	8.28	75	8.76

The Table reports the distribution of all the other birth defects besides CL/P observed in the associated cases. Minor anomalies angioma, neonatal tooth, gum/tongue anomaly, undescended testes, nevus, micorgnathia and other minor anomalies. Other anomalies include other eye anomalies, anomalies of the neck, nose, trachea, skull shape and muscle. Also included are other unspecified birth defects (1 birth defect in the control group and 17 birth defects in the treatment group). The distributions of birth defect types between the treatment and control groups are different at p = 0.027 based on a chi-square test of independence. However, the test result should be viewed with caution given the very low frequencies of several birth defect types.

The study hospitals were affiliated with the Latin American Collaborative Study of Congenital Malformations (ECLAMC), which is a birth defect surveillance and research program throughout South America [29]. ECLAMC involves collaboration with pediatricians who monitor birth defect occurrence in their hospitals and collect birth record data on affected and unaffected infants. The ECLAMC pediatricians were responsible for identifying and enrolling infants into the study and providing the intervention.

The intervention sample included 663 infants enrolled within 48 hours after birth and before hospital discharge. The sample represents 94% of eligible potential subjects (707 cases). The other potential subjects did not consent to the study. Parental consent was obtained from each child's parent(s) before participation.

The control sample included 456 infants who represent 91% of potential control subjects (499 cases). The non-intervened control group had received the medical care available in the community, which is unmeasured but is thought to vary between the various communities included in the study and to be less intensive and systematic on average than the study intervention. The study pediatricians identified the control group from the ECLAMC and hospital birth records and conducted phone and/or in-person interviews with parents to obtain data on infant survival and hospitalizations. The pediatricians also abstracted related information from the infants' hospital charts.

As described below, the intervention started at the 7^th ^day of life. Therefore, all infants who died before the 7^th ^day were excluded from the analysis as they had not received the intervention as defined in the protocol, resulting in a final sample of 543 infants in the intervention group and 372 controls for the survival analysis.

Table 2 presents a comparison of baseline health and other relevant characteristics between the study populations and enrolled samples by intervention status. There were no statistically significant differences between the study populations and samples, suggesting no sample selection bias due to parental consenting into the study. The sample is geographically and socioeconomically diverse, which enhances its representativeness of the infant populations in the study countries. The majority of infants in the study countries are thought to be born in healthcare institutions [30]. The WHO reports that rates of births attended by skilled health personnel, most of which are expected to have occurred in healthcare institutions, ranged from about 61% in Bolivia to ~100% in Chile in 2003-2005 [31]. The study results are generalizable to in-hospital born infants, who represent the majority of infants in the study populations.

**Table 2:** Distribution of Study Variables in the Population and Study Samples by Intervention Group

Variable	Control Group			Intervention Group		
	
	Population	Total Sample	Sub-sample with Mortality Outcome	Population	Total Sample	Sub-sample with Mortality Outcome
Overall Sample Size	499	456	441	707	663	602
Maternal Age	25.78 (6.89)	25.77 (6.94)	25.79 (6.89)	26.37 (6.92)	26.30 (6.86)	26.29 (6.86)
Number of prenatal visits	5.58 (3.01)	5.65 (2.95)	5.66 (2.94)	4.51 (3.51)	4.56 (3.51)	4.67 (3.51)
Maternal chronic illness (N)	469	431	419	702	659	598
No (%)	395(84.2)	358 (83.1)	347 (82.8)	548 (78.1)	516 (78.3)	471 (78.8)
Yes (%)	74(15.8)	73 (16.9)	72 (17.2)	154 (21.9)	143 (21.7)	127 (21.2)
Ethnic Ancestry (N)	465	427	416	691	652	592
Black (%)	51(11.0)	51 (11.9)	50 (12.0)	81 (11.7)	77 (11.8)	68 (11.5)
Native (%)	348(74.8)	313 (73.3)	303 (72.8)	523 (75.7)	492 (75.5)	449 (75.8)
Other (%)	66(14.2)	63 (14.8)	63 (15.1)	87 (12.6)	83 (12.7)	75 (12.7)
Birth weight (grams)	2887.5 (788.5)	2,863.5 (797.8)	2870.8 (803.7)	2881.9 (740.3)	2,892.9(726.7)	2871.3 (732.2)
Gestational age (weeks)	38.3 (3.4)	38.3 (3.4)	38.3 (3.5)	38.5 (3.1)	38.5 (3.1)	38.4 (3.2)
Time of cleft diagnosis (N)	495	454	439	701	657	596
Prenatal (%)	80 (16.2)	76 (16.7)	73 (16.6)	174 (24.8)	161 (24.5)	145 (24.3)
Other (%)	415(83.8)	378 (83.3)	366 (83.4)	527 (75.2)	496 (75.5)	451 (75.7)
Sex (N)	491	450	437	705	661	601
Male (%)	275(56.0)	250 (55.6)	245 (56.1)	394 (55.9)	372 (56.3)	338 (56.2)
Female (%)	216(44.0)	200 (44.4)	192 (43.9)	311 (44.1)	289 (43.7)	263 (43.8)
Multiple birth (N)	496	455	440	707	663	602
No (%)	484(97.6)	443 (97.4)	428 (97.3)	688 (97.3)	646 (97.4)	586 (97.3)
Yes (%)	12 (2.4)	12 ( 2.6)	12 (2.7)	19 (2.7)	17 ( 2.6)	16 (2.7)
Associated Status (N)	499	456	441	707	663	602
Isolated (%)	361(72.3)	325 (71.3)	313 (71.0)	478 (67.6)	451 (68.0)	405 (67.3)
Associated (%)	138(27.7)	131 (28.7)	128 (29.0)	229 (32.4)	212 (32.0)	197 (32.7)
Type of Cleft (N)	496	455	441	706	663	602
Palate only (%)	129(26.0)	121 (26.6)	118 (26.8)	197(27.9)	181 (27.3)	163 (27.1)
Lip only (%)	87(17.5)	77 (16.9)	75 (17.0)	120(17.0)	111 (16.7)	101 (16.8)
Lip and Palate (%)	280(56.5)	257 (56.5)	248 (56.2)	389(55.1)	371 (56.0)	338 (56.2)
Type of Delivery (N)	492	451	436	707	663	602
Cesarean (%)	184(37.4)	168 (37.3)	163 (37.4)	280(39.6)	258 (38.9)	238 (39.5)
Other (%)	308 (62.6)	283 (62.7)	273 (62.6)	427 (60.4)	405 (61.1)	364 (60.5)

The Table includes the distribution of study variables in the study population, study sample and sample with mortality data. Means and standard deviations (in parentheses) are reported for continuous variables. Counts and percentages (in parentheses) are reported for categorical variables.

The following comparisons were examined within each of the intervention and control groups: 1) population versus study sample and 2) study sample versus sample with mortality data.

The differences in the number of observations in the subsamples with mortality outcome in this table and those included in mortality analysis in Table 1 is due to limiting the mortality analysis to those who were alive on the 7^th ^day after birth. The subsamples with mortality outcome in Table 6 include all consented infants with known mortality outcome including those who died before the 7^th ^day of life since the goal is to check for any characteristics that are related to dropping out of the study resulting in unknown mortality status.

The study was approved by the University of Iowa IRB (protocol ID 200109029).

### Neonatal Pediatric Care Intervention

The intervention consisted of four weekly visits with the study pediatricians between the 7^th ^and 28^th ^day of life which involved systematic evaluation of the infant's health, providing pediatric care, referral to specialized care as needed and educating parents in feeding practices and optimal infant care and monitoring. First, the pediatricians screened infants for eligibility and enrolled those who are eligible into the study within the first 48 hours and before hospital discharge. As part of this screening, the pediatricians advised mothers about needed healthcare and optimal feeding and homecare practices following ECLAMC'S routine ORIENT program, which provides affected families with cleft- related information and orients them to available healthcare resources [29]. The control families had also received the ORIENT program information at this period.

Once a week, the study pediatricians monitored and assessed the health of the intervention group infants during in-person visits to the pediatricians' practices. The pediatricians conducted home visits when possible with families who missed the weekly visits. The pediatricians evaluated changes in weight, length, head circumference, feeding methods and medical complications such as hyperbilirubinemia or infection. After the examination, the pediatricians referred the infants for further intervention to specialized professionals as needed. The pediatricians assessed the need for hospitalizing infants who showed no weight gain between visits and for more frequent follow-up than the weekly visit schedule for these infants or infants with other medical complications. The study protocol required the pediatricians to hospitalize infants who show more than ten percent weight decrease between visits and to evaluate the need for hospitalizing infants with ten percent or less weight decrease based on the infant's health, medical complications and standard pediatric judgment. The study protocol required the pediatricians to re-emphasize at each visit the recommendations and instructions to parents on feeding practices and optimal homecare. The control group did not receive the weekly pediatric visits provided to the intervention group.

### Study Outcome Measures

The study assessed the intervention effect on neonatal mortality between the 7^th ^and 28^th ^day of life and on hospitalization days during this period among surviving infants, both pooling and stratifying by CL/P associated status. These outcomes were measured beginning on the 7^th ^day of life given that the pediatric intervention started on that day. We also report the overall neonatal mortality rates between the first and 28^th ^day of life given that such estimates are rare in South America.

The study pediatricians assessed survival and hospitalizations using the same study questionnaires at all hospitals during their visits with the intervention group and interviews with the control group. Further details of data collection and management are described elsewhere [26].

### Statistical Analysis

Given the non-randomized design of the study, we assessed the intervention effects on the study outcomes adjusting for relevant baseline characteristics including infant's cleft type (cleft lip, cleft palate, cleft lip with palate), birth weight, gestational age, sex and ethnic ancestry, maternal age, number of prenatal visits, maternal chronic illness, multiple birth status, cesarean-delivery and prenatal CL/P diagnosis. The regression models included country fixed effects in order to account for country differences. As a reference, we also estimated regressions that did not adjust for baseline characteristics but only included the treatment and country indicators. ECLAMC pediatricians routinely measure these baseline characteristics through interviews with the mothers after delivery and before hospital discharge and through hospital record abstraction. Table 3 describes the baseline characteristics in the study intervention and control groups. There were no significant differences in these characteristics between the intervention and control groups except for a greater number of prenatal visits and greater birth weight among the isolated control group, and a higher rate of prenatal CL/P diagnosis among the isolated intervention group.

**Table 3:** Distribution of Baseline Characteristics in the Intervention and Control Groups Included in the Mortality Analysis

	Overall		Isolated		Associated	
**Variable**	**Intervention** **N = 543**	**Control** **N = 372**	**Intervention N = 394**	**Control** **N = 305**	**Intervention** **N = 149**	**Control** **N = 67**

Maternal Age	26.23 (6.83)	25.50 (6.67)	25.88 (6.58)	25.40 (6.53)	27.14 (7.39)	25.92 (7.30)

Number of prenatal visits	4.74*** (3.54)	5.87 (2.91)	4.47*** (3.56)	5.95 (2.87)	5.47 (3.39)	5.51 (3.09)

Maternal chronic illness						

No (%)	425 (78.7)	291(82.4)	315 (80.6)	237(82.6)	110 (73.8)	54(81.8)

Yes (%)	115 (21.3)	62 (17.6)	76 (19.4)	50 (17.4)	39 (26.2)	12(18.2)

Ethnic Ancestry						

Black (%)	57(10.7)	31 (8.8)	41 (10.5)	26 (9.1)	16 (11.0)	5 (7.7)

Native American (%)	407 (76.3)	262(74.4)	296 (76.1)	209(72.8)	111 (76.6)	53(71.5)

Other (%)	70 (13.1)	59 (16.8)	52 (13.4)	52 (18.1)	18 (12.4)	7 (10.7)

Birth weight (grams)	2,976.7* (648.6)	3,046.6 (670.3) (797.75)	3,031.9** (618.1)	3,123.8 (631.0)	2,831.0 (704.8)	2,694.8 (733.5)

Gestational age (weeks)	38.75 (2.86)	38.92 (2.74)	38.89* (2.80)	39.19 (2.51)	38.38 (2.97)	37.71 (3.37)

Time of cleft diagnosis						

Prenatal (%)	116 (21.6) ***	38 (10.3)	64 (16.4)***	24 (7.9)	52 (35.4)**	14(21.2)

Other (%)	421 (78.4)	332(89.7)	326 (83.6)	280(92.1)	95 (64.6)	52 (78.8)

Sex						

Male (%)	307 (57.3)	213(57.3)	222 (56.4)	178(58.4)	85 (57.1)	35(52.2)

Female (%)	236 (43.5)	159(42.7)	172 (43.7)	127(41.6)	64 (43.0)	32(47.8)

Multiple birth						

No (%)	529 (97.4)	361(97.3)	381(96.7)	295(97.0)	148 (99.3)	66 (98.5)

Yes (%)	14 ( 2.6)	10 (2.7)	13 (3.3)	9 (3.0)	1 (0.7)	1 (1.5)

Associated Status						

Isolated (%)	394 (72.6)	305(82.0)	-	-	-	-

Associated (%)	149 (27.4)	67 (18.0)	-	-	-	-

Type of Cleft						

Palate only (%)	142 (26.2)	92 (24.7)	83 (21.1)	66 (21.6)	59 (39.6)	26(38.8)

Lip only (%)	96 (17.7)	67 (18.0)	88 (22.3)	60 (19.7)	8 (5.4)	7 (10.5)

Lip and Palate (%)	305 (56.2)	213(57.3)	223 (56.6)	179(58.7)	82 (55.0)	34(50.8)

Type of Delivery						

Cesarean (%)	202 (37.2)	127(34.5)	132 (33.5)	94 (31.2)	70 (47.0)	33(49.3)

Other	341(62.8)	241(65.5)	262(66.5)	207(68.8)	79 (53.0)	34(50.8)

The Table includes the distribution of study variables in the study intervention and control groups that are included in the mortality analysis. Means and standard deviations (in parentheses) are reported for continuous variables. Counts of observations and percentages (in parentheses) are reported for categorical variables. Significant differences between intervention and control groups are indicated with * at p < 0.1, ** at p < 0.05 and *** at p < 0.01.

The mortality outcome model was estimated by logistic regression. The hospitalization day model was estimated by zero-inflated negative binomial regression in order to account for the large percentage of zero hospitalizations. The variance-covariance matrices were estimated with a Huber-type estimator that is robust for within-hospital clustering [32].

The intervention effects were estimated pooling and stratifying by CL/P associated status given the expected effect differences by the presence of other birth defects. The models for total and associated samples included the number of birth defects other than CL/P as a covariate in order to account for the severity of associated status.

About 6.8% of the sample were lost to follow-up and had no observable mortality outcomes. Table 2 presents a comparison between the enrolled sample and the sample with known mortality status. There were no significant differences in baseline characteristics between these samples for both the intervention and control groups, suggesting no sample selection bias. Therefore, we did not adjust the mortality and hospitalization models for sample selection.

## Results

### Neonatal Mortality

Table 4 reports the mortality rates between the 7^th ^and 28^th ^day in the intervention and control groups and the odds ratios (ORs) for the intervention effect from the logistic regression. Table 5 reports the ORs for all model variables.

**Table 4:** Mortality between 7^th ^and 28^th ^day in the in intervention and control groups

Group	Mortality Rate (%)		Effect of Intervention (Odds Ratio)	
	
	Intervention	Control	Unadjusted	Adjusted
Overall Sample	6.08	4.84	1.28 [0.71,2.32]	1.17 [0.43,3.23]

Isolated	2.03	1.31	1.48 [0.49,4.49]	2.34 [0.64,8.64]

Associated	16.78	20.90	0.76 [0.36,1.61]	0.96 [0.34,2.66]

The Table reports the neonatal mortality rates in the study intervention and control groups and the odds ratios for the intervention effects on mortality. The unadjusted model includes indicators for the country of birth as covariates. The 95% confidence intervals (CI) of the odds ratios are in brackets.

**Table 5:** Odds Ratios for the Effects of the Intervention and Baseline Characteristics on Mortality between the 7^th ^and 28^th ^day

	Total Sample		Isolated Sample		Associated Sample	
Study intervention group	1.28 [0.71,2.32]	1.17 [0.43,3.23]	1.48 [0.49,4.49]	2.34 [0.64,8.64]	0.76 [0.36,1.61]	0.96 [0.34,2.66]
Bolivia	2.64***	5.98***	4.70***	5.54	12.61***	7.48***
	[1.59,4.37]	[2.23,16.04]	[1.48,14.91]	[0.51,59.72]	[6.41,24.78]	[2.25,24.88]
Brazil	1.17	0.98	1.54	1.69	0.89	2.46
	[0.50,2.76]	[0.28,3.39]	[0.30,7.92]	[0.20,13.96]	[0.33,2.46]	[0.60,10.21]
Chile	0.58	0.45	0.51	0.35	0.46	0.53
	[0.21,1.61]	[0.13,1.52]	[0.06,4.24]	[0.02,6.37]	[0.16,1.35]	[0.14,2.02]
Colombia	1.34	1.46	1.65	0.27	2.33**	4.75
	[0.68,2.63]	[0.46,4.63]	[0.26,10.59]	[0.03,2.72]	[1.07,5.04]	[0.69,32.76]
Ecuador	0.61	3.27*	0.74	1.36	1.13	4.00*
	[0.27,1.34]	[0.97,10.95]	[0.07,7.68]	[0.03,73.00]	[0.42,3.04]	[0.88,18.24]
Venezuela	1.79**	1.44	3.56**	1.45	2.18**	3.69*
	[1.08,2.96]	[0.42,4.98]	[1.10,11.55]	[0.28,7.58]	[1.15,4.16]	[0.78,17.44]
Maternal age (years)		0.99		1.02		0.98
		[0.94,1.04]		[0.87,1.20]		[0.93,1.03]
Number of prenatal visits		1.00		0.97		0.98
		[0.86,1.16]		[0.68,1.40]		[0.85,1.12]
Maternal chronic illnesses		0.78		0.31		1.08
		[0.34,1.78]		[0.02,5.71]		[0.44,2.62]
African ancestry		32.19***		7.74*		
		[2.53,410.06]		[0.81,73.92]		
Native ancestry		22.03**		2.39		4.41**
		[1.61,302.34]		[0.18,32.35]		[1.42,13.74]
Prenatal diagnosis of cleft		0.79		0.09*		0.77
		[0.24,2.59]		[0.01,1.50]		[0.25,2.38]
Associated status		6.60***				
		[1.88,23.25]				
Birth weight (gm)		0.998***		0.998***		0.999**
		[0.998,0.999]		[0.997,0.999]		[0.998,0.999]
Gestational age (weeks)		0.92		0.78*		0.99
		[0.80,1.07]		[0.60,1.00]		[0.84,1.17]
Female		0.83		0.96		1.09
		[0.38,1.85]		[0.19,4.94]		[0.39,3.07]
Multiple birth		0.22*		0.32		
		[0.05,1.05]		[0.04,2.50]		
Cleft lip only		1.26		7.63*		0.77
		[0.42,3.83]		[0.95,61.23]		[0.14,4.25]
Cleft lip with palate		0.89		6.71*		0.55
		[0.46,1.73]		[0.83,54.30]		[0.26,1.16]
Number of non-cleft Malformations		1.60***				1.53***
		[1.26,2.04]				[1.23,1.91]
Cesarean delivery		1.56		0.89		2.25
		[0.58,4.23]		[0.24,3.28]		[0.72,6.98]

*N*	915	776	699	591	216	185

The Table reports the odds ratios for the effects of the intervention and baseline characteristics on mortality between the 7th and 28th day of life. 95% Confidence intervals are in brackets. *, ** and *** indicate p < 0.1, < 0.05 and < 0.01, respectively.

There were no significant differences in the 7^th^-28^th ^day mortality rates between the intervention and control groups, both pooled and stratified by associated status. In the total sample, the mortality rate was 6.1% in the intervention group versus 4.8% in the control group. In the isolated group, the mortality rates were 2% versus 1.3% in the intervention versus control groups, respectively. In the associated group, the mortality rates were 16.8% versus 20.9% in the intervention and control groups, respectively.

The overall neonatal mortality rates between the first and 28^th ^day of life were 15.3% and 19.7% in the intervention and control groups, respectively. The overall neonatal mortality rates were 4.7% and 3.8% in the isolated intervention and control groups, respectively, and 37.1% and 58.6% in the associated intervention and control groups, respectively.

### Neonatal Hospitalization Days

Table 6 reports hospitalization days and the intervention effects on hospitalization days between the 7^th ^and 28^th ^days of life among the infants who survived the neonatal period. Table 7 reports the effects of all model variables.

**Table 6:** Number of hospitalization days between 7^th ^and 28^th ^day among surviving infants and intervention effects

	Number of hospitalization days		Intervention effect	
	**Intervention**	**Control**	**Unadjusted**	**Adjusted**

Overall Sample	1.92 (4.73)	3.09 (6.19)	-1.03** (0.43)	-0.63* (0.37)

Isolated	1.55 (4.41)	1.91 (4.61)	-0.36 (0.40)	0.023 (0.26)

Associated	3.39 (5.64)	10.34 (9.11)	-6.80*** (1.75)	-6.06*** (2.08)

The Table reports the average number of hospitalization days between the 7^th ^and 28^th ^day of life in the intervention and control groups and the intervention effects on hospitalization days. The unadjusted model includes indicators for the country of birth as covariates. *, ** and *** indicate p < 0.1, < 0.05 and < 0.01, respectively. The standard deviations of the number of days hospitalized and the standard errors of the intervention effects are in parentheses.

**Table 7:** Effects of the Intervention and Baseline Characteristics on Hospitalization Days between the 7^th ^and 28^th ^Day of Life

	Total Sample		Isolated Sample		Associated Sample	
	
Study intervention group	-1.03**	-0.63*	-0.36	0.023	-6.80***	-6.06***
	(0.43)	(0.37)	(0.40)	(0.26)	(1.75)	(2.08)
Bolivia	0.18	-0.032	0.45	-0.30	7.67***	17.7**
	(0.77)	(0.56)	(0.58)	(0.33)	(2.15)	(8.63)
Brazil	-0.42	-0.14	-0.73*	-0.28	-0.54	-0.47
	(0.63)	(0.64)	(0.41)	(0.42)	(1.05)	(3.03)
Chile	-0.53	-0.49	-0.15	-0.24	-3.51***	-3.79**
	(0.91)	(0.69)	(0.84)	(0.48)	(1.05)	(1.61)
Colombia	-1.41***	-1.33***	-0.90*	-0.96***	-2.40	-3.40**
	(0.52)	(0.33)	(0.50)	(0.19)	(1.85)	(1.54)
Ecuador	-1.39**	-0.36	-0.82*	-0.34	-2.89	0.17
	(0.58)	(0.59)	(0.45)	(0.41)	(1.99)	(5.53)
Venezuela	-1.80***	-1.21***	-1.48***	-1.00***	-2.87	-1.07
	(0.66)	(0.43)	(0.37)	(0.23)	(2.87)	(2.98)
Maternal age (years)		-0.028		-0.026		0.14
		(0.022)		(0.019)		(0.10)
Number of prenatal visits		0.022		0.048		0.029
		(0.044)		(0.035)		(0.26)
Maternal chronic illnesses		0.76		0.72*		-0.95
		(0.46)		(0.41)		(1.54)
African ancestry		-0.19		-0.34		2.86
		(0.43)		(0.28)		(3.13)
Native ancestry		0.49		0.42		0.66
		(0.66)		(0.47)		(2.72)
Prenatal diagnosis of cleft		-0.24		-0.48*		-0.69
		(0.52)		(0.29)		(2.11)
Associated status		2.16**				
		(1.05)				
Birth weight (gm)		-0.0009***		-0.00079***		-0.000056
		(0.00029)		(0.00024)		(0.0019)
Gestational age (weeks)		-0.21***		-0.13***		-0.52
		(0.072)		(0.050)		(0.42)
Female		-0.016		0.11		-0.99
		(0.31)		(0.22)		(1.29)
Multiple birth		-2.30**		-1.00		
		(1.10)		(0.70)		
Cleft lip only		-1.51***		-1.33***		-1.95
		(0.37)		(0.25)		(3.25)
Cleft lip with palate		-0.63*		-0.53*		-0.33
		(0.36)		(0.31)		(2.13)
Number of non-cleft Malformations		0.27				0.72
		(0.23)				(0.54)
Cesarean delivery		0.81**		0.65*		-0.049
		(0.38)		(0.34)		(1.18)
*N*	772	660	638	543	134	117

The Table reports the effects of the intervention and baseline characteristics on hospitalization days between the 7^th ^and 28^th ^day of life. *, ** and *** indicate p < 0.1, < 0.05 and < 0.01, respectively. The standard errors are in parentheses.

The intervention group had fewer neonatal hospitalization days on average compared to the control group (1.9 versus 3.1 days). In the multivariate model, the intervention reduced hospitalization days by about 0.6 days in the total sample (p = 0.089). There was no significant difference in hospitalization days between the isolated intervention and control groups (1.6 versus 1.9 days, respectively). In contrast, there were significantly fewer hospitalization days in the intervention group with associated CL/P compared to the control group (3.4 versus 10.3 days, respectively). In the multivariate model, the intervention reduced hospitalization days in the associated group by about 6 days (p < 0.001).

Table 8 reports the distribution of reasons for hospital readmission for the treatment group; data is not available on hospital readmission reasons for the control group. About 18% of readmissions were due to respiratory problems and 16% were due to weight loss problems. About 12% were due to feeding or nutrition problems. Another 10% were due to surgery including cleft repair or any other surgery, and another 10% were due to icterus syndromes.

**Table 8:** Reasons for Hospital Readmission among Treatment Group

	Frequency	Percent
Respiratory problems	9	18.0

Weight loss or other weight problems	8	16.0

Nutrition/feeding problems	6	12.0

Icterus syndromes	5	10.0

Surgery	5	10.0

Sepsis	2	4.0

Other infection and fever	3	6.0

Other	6	12.0

Not specified	6	12.0

TOTAL	50	100.0

The Table reports the frequencies of the reasons for hospital readmission for infants in the treatment group who survived the neonatal period.

### Effects of Baseline Factors on Mortality and Hospitalizations

We describe below the significant effects of main baseline infant and maternal characteristics on mortality and hospitalization days between the 7^th ^and 28^th ^day of life (see detailed results in Tables 5 and 7). African and native infant ancestry, as reported by the mother, increased mortality risk by about 32 and 22 times in the total sample, respectively, compared to other ancestries. Prenatal CL/P diagnosis decreased mortality risk by about 0.1 times in the isolated group (marginally significant). Higher birth weight reduced mortality significantly both pooling and stratifying by associated status - a 300-gram birth weight decreased mortality risk by about 0.7 times in the total sample. In the isolated group, infants with cleft lip only and cleft lip with palate had higher mortality risks by about 7.6 and 6.7 times, respectively, compared to infants with cleft palate only. Finally, the number of other malformations besides CL/P increased mortality risk in the associated group by 1.5 times per malformation.

Several factors had significant effects on hospitalization days in the total sample, primarily due to effects on the isolated CL/P group. Birth weight and gestational age each reduced hospitalization days by about one day per 1000 grams and five gestational weeks, respectively. Infants with cleft lip only and with cleft lip with palate in the total sample had fewer hospitalization days by about 1.5 and 0.6 days, respectively, compared to infants with cleft palate only. Also, infants born through cesarean delivery had longer hospitalization by about one day. Similar effects were seen on hospitalization in the isolated group, in addition to a small decrease in hospitalizations for cases diagnosed prenatally (half a day on average; marginally significant) and an increase in hospitalization days among those whose mothers had chronic illnesses during pregnancy. (0.7 days on average).

## Discussion

The study finds that early systematic pediatric care may significantly reduce the neonatal morbidity of infants with associated CL/P in South America who survive the neonatal period, for whom the intervention reduced hospitalization stay by about six days. This suggests that systematic pediatric care may significantly reduce inpatient costs of associated cases. In the US, first year of life healthcare costs for infants with associated CL/P exceed those of unaffected children by more than 25 times [16]. As studies evaluate the effectiveness of primary prevention programs for CL/P such as folic acid supplementation [33], tertiary prevention programs are essential for reducing the health risks and economic burden of CL/P for affected individuals and families. About half of the neonatal hospital readmissions in the treatment group were due to respiratory, weight loss, or feeding and nutrition problems. This highlights the adverse oral-cleft effects on early health and the importance of providing adequate advice and training to parents before hospital discharge after birth about optimal household approaches to reduce these health problems, especially the feeding problems.

There was a markedly larger benefit from the intervention for reducing hospitalizations in the associated than the isolated group. This is likely due to the greater health needs and more frequent and longer hospitalizations among the associated group that may be responsive to the intervention. On average, infants in the associated control group were hospitalized for about 10 days between the 7^th ^and 28^th ^days of life, compared to about 2 days for the isolated control group. This highlights the significantly elevated health problems among the associated group. This differential effect between the isolated and the associated group is consistent with the theory that the intervention is more relevant on average for infants with greater health needs that can be identified and addressed by the intervention.

Congenital anomalies are a leading cause of neonatal mortality and morbidity worldwide [34]. Despite substantial progress in achieving the Millennium Development Goals in reducing under-five mortality worldwide, there has been far less improvement in neonatal mortality [34],[35]. The study suggests that increasing access to systematic pediatric care alone may not reduce the contribution of congenital anomalies to neonatal mortality. A potential contributor to this result is that CL/P impose a larger burden on infant survival immediately after birth and during the first few days of life during which infants typically are hospitalized. After that, mortality risks drop significantly in both the isolated and associated groups. About 62% and 76% of deaths in the isolated group occurred by the 7^th ^and 8^th ^day of life, respectively. Similarly, 75% of deaths in the associated group occurred by the 7^th ^day of life. The study's intervention began on the 7^th ^day of life given that at-risk infants generally remain hospitalized during the first few days of life and receive standard in-patient pediatric care. Further, CL/P in the associated groups are associated with major structural limitations and malformations including cardiac and neural tube abnormalities that may reduce the intervention effects on survival.

The study is among the first studies to estimate mortality rates among infants with CL/P in South America. The results provide evidence that neonatal mortality and morbidity are very high among infants with associated CL/P in South America and that CL/P mortality rates are higher in South American countries than the US [11-13]. Hujoel et al (1992) report that neonatal mortality rates of infants with CL/P in the state of Washington in 1984-1988 were about 1.6% in the isolated group and 31.2% in the associated group [13]. Druschel et al. (1996) report that first-year mortality rates of infants with CL/P in New York State between 1983 and 1991 were about 1% in the isolated group and 23.5% in the associated group [11].

The study finds significant ethnic disparities in neonatal mortality of infants with CL/P in South America, with significantly higher risks among infants with African and Native ethnic ancestry compared to other ancestries. Racial disparities in infant health outcomes have been documented in several of the study countries [36], consistent with the study findings. For example, neonatal and infant mortality rates in Brazil are twice as high among children of black or mixed race women as whites [37]. Identifying and targeting the contributing factors is needed for reducing disparities. Cleft lip with/without palate, lower birth weight, shorter gestational age and the presence of multiple malformations besides CL/P are additional risk factors for neonatal mortality risk that can be routinely evaluated to identify infants with CL/P who are at higher mortality risks. The greater mortality risk among cases with cleft lip compared to cleft palate alone is surprising, especially in the case of cleft lip alone. It is unclear what factors are driving this result. It is possible that cleft lip may be associated with greater parental stress and anxiety in the neonatal period compared to cleft palate alone, which may have adverse effects on the infant's household environment and care. More research is needed to identify the reasons for this increase in mortality risks.

One study limitation is the use of a non-randomized control group. As mentioned above, the investigators considered randomization to be unethical. However, the similarity between the study intervention and control groups on most baseline characteristics supports the utility of identifying a natural control group born in a very close period to the intervention group in the same communities when randomization is infeasible. Furthermore, adjusting for the relevant baseline characteristics is expected to account for any differences between the two groups that may bias the treatment effect estimates.

The study provides an intervention model and research design that may be implemented in other studies to evaluate the effects of early systematic pediatric care on the neonatal survival and health of infants born with other common and burdensome birth defects. Such birth defects include neural tube defects, congenital heart anomalies and Down Syndrome which also have high mortality and morbidity especially in less developed countries [38].

## Conclusion

The study finds that early systematic pediatric care significantly reduces neonatal hospitalization days for infants with associated CL/P who survive the neonatal period. Given the large health returns, the reduced hospitalization costs and the low cost of providing systematic pediatric care, improving the access of these infants to systematic pediatric care programs is likely to be a cost-effective public policy intervention, although a formal evaluation of the cost-effectiveness of the study's intervention is needed to accurately compare the added health value to the costs at the population-level. Future studies are needed to assess whether the effects of early systematic pediatric care may vary by socioeconomic, clinical and demographic characteristics, and to identify the effects of such care beyond the neonatal period. Such studies will identify infant groups who may benefit most from pediatric care in order to design cost-effective policies and interventions to improve access to and supply of pediatric care, and to maximize the returns of these initiatives. Focused interventions are particularly needed in resource-constrained environments such as in the study countries. Other studies have found larger prenatal care effects on birth weight among pregnancies with higher fetal health risks [39]. Pediatric care may be more effective in the groups with the largest mortality and morbidity risks through identifying and addressing the higher risk factors.

## Competing interests

The authors declare that they have no competing interests.

## Authors' contributions

Drs. EEC and JCM conceived and designed the study. Drs. GLW, VC, MR, GD, LJ, JSL and IMO co-designed the study and were involved in writing study protocols, manual of operations and data collection questionnaires. Drs. GLW, JCM and EEC and Mr. NG oversaw the study implementation. Drs. GLW and HC and Mr. MK designed and conducted the statistical analysis. All authors contributed to writing the manuscript. All authors have read and approved the final manuscript.

## Pre-publication history

The pre-publication history for this paper can be accessed here:

http://www.biomedcentral.com/1471-2431/11/121/prepub
